# Ventricular Angiography: A Forgotten Diagnostic Tool?

**DOI:** 10.3390/diagnostics14131434

**Published:** 2024-07-05

**Authors:** Georgiana Pintea Bentea, Brahim Berdaoui, Sophie Samyn, Marielle Morissens, Jose Castro Rodriguez

**Affiliations:** Department of Cardiology, CHU Brugmann, 1020 Brussels, Belgium

**Keywords:** arrhythmogenic right ventricular cardiomyopathy, right ventricular angiography, diagnostic image

## Abstract

A 76-year-old male patient presented to the emergency room with acute decompensated right heart failure and presyncope episodes. Upon admission, his electrocardiogram (ECG) showed sustained monomorphic ventricular tachycardia at 180 bpm, which was electrically cardioverted, and the patient was subsequently admitted to the intensive care unit. The echocardiography showed a very dilated right ventricle (RV) with global systolic dysfunction and akinetic anterior and lateral walls. The coronary angiography was normal. The cardiac magnetic resonance showed signs of fibro-fatty replacement of the RV myocardium. Furthermore, the ECG after cardioversion showed inverted T waves and an epsilon wave in V1–V3 leads and late potentials by signal-averaged ECG. As such, a diagnosis of arrhythmogenic right ventricular cardiomyopathy (ARVC) was suspected. However, he presented no familial history of ARVC, was 76 years of age at the time of diagnosis and was asymptomatic until now. Given these considerations, we performed a right ventricular angiography which showed dilatation of the RV with akinetic/dyskinetic bulging, creating the “pile d’assiettes” image suggestive of ARVC. In the case of this patient, the RV angiography contributed to establish a diagnosis of ARVC with a very late presentation, to our knowledge the latest presentation in terms of age described in the literature.

**Figure 1:**
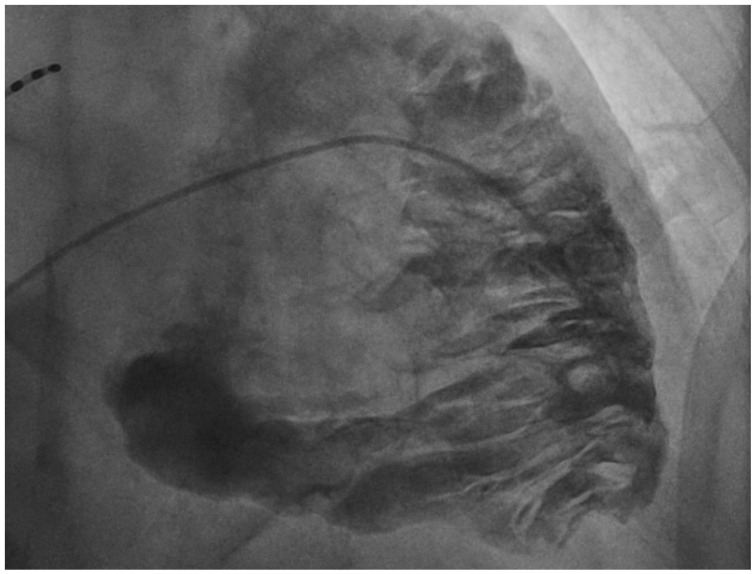
Right ventricular angiography showing dilatation of the right ventricle with akinetic/dyskinetic bulgings, creating the “pile d’assiettes” image suggestive of arrhythmogenic right ventricular cardiomyopathy. A 76-year-old male patient with past history of chronic obstructive pulmonary disease, right iliac artery stenting, dyslipidaemia, high blood pressure and previous smoking, presented to the emergency department of our institution with chest tightness, signs and symptoms of acute decompensated right heart failure and presyncope episodes. Upon admission, his electrocardiogram (ECG) showed sustained monomorphic ventricular tachycardia at 180 bpm, with left bundle branch morphology and a superior axis, which was electrically cardioverted, and the patient was subsequently admitted to the intensive care unit. The troponin (601 ng/L) and NTproBNP (24,019 ng/L) levels were elevated, the echocardiography showed a very dilated right ventricle (RV) (a diameter of the RV outflow track in end-diastole of 44 mm in parasternal short axis view) with global systolic dysfunction and akinetic anterior and lateral walls. The coronary angiography was normal. The cardiac magnetic resonance (CMR) confirmed global RV dysfunction with regional akinesia and aneurysms and showed signs of fibro-fatty replacement of the RV myocardium. There were no left ventricular abnormalities identified by echocardiography or CMR. Furthermore, the ECG after cardioversion showed inverted T waves and an epsilon wave in V1-V3 leads, in the absence of right bundle branch block and late potentials by signal-averaged ECG. As such, the patient presented criteria for the diagnosis of arrhythmogenic right ventricular cardiomyopathy (ARVC) as described by the 2020 “Padua Criteria” for the diagnosis of ARVC [1], recently revised by the 2024 European Task Force consensus report [2]. However, he presented no familial history of ARVC, was 76 years of age at the time of diagnosis and was asymptomatic until now. Given these considerations, we performed a right ventricular angiography which showed dilatation of the RV with akinetic/dyskinetic bulging, creating the “pile d’assiettes” image suggestive of ARVC (Video S1). The RV angiography has a diagnostic specificity of more than 90% [3] and was considered the gold standard diagnostic exam for ARVC, particularly before the era of advancements in cardiac magnetic resonance imaging [4], while more recent studies on this subject are sparse. In the case of this patient, the RV angiography contributed to establish a diagnosis of ARVC with a very late presentation, to our knowledge the latest presentation in terms of age described in the literature. Subsequently, the patient was implanted with a defibrillator, and his family benefited from screening.

## Supplementary Materials

The following supporting information can be downloaded at: https://www.mdpi.com/article/10.3390/diagnostics14131434/s1, Video S1: Right ventricular angiography showing dilatation of the right ventricle with akinetic/dyskinetic bulgings, creating the “pile d’assiettes” image suggestive of arrhythmogenic right ventricular cardiomyopathy.
